# Investigation of the Effects of Antibiotic Application on the Intestinal Flora in Elderly Hypertension Patients with Infectious Diseases

**Published:** 2018-03

**Authors:** Changhai SU, Yang LIU, Haiwen ZHANG, Bin XIAO, Te’er BA

**Affiliations:** 1. Dept. of Clinical Pharmacy, Ordos Central Hospital, Ordos, Inner Mongolia, P.R. China; 2. Dept. of Internal Medicine, Ordos Central Hospital, Ordos, Inner Mongolia, P.R. China; 3. Dept. of Clinical Laboratory, Ordos Central Hospital, Ordos, Inner Mongolia, P.R. China

**Keywords:** Antibiotic, Intestinal flora, Hypertension patients, Infectious diseases, Bacteriological analysis

## Abstract

**Background:**

This study aimed to investigate the effects of antibiotic application on the intestinal flora in elderly hypertension patients with infectious diseases.

**Methods:**

A total of 2350 infected patients treated in Ordos Central Hospital (Inner Mongolia, China) from January 2010 to August 2016 were retrospectively analyzed and 790 healthy hypertension patients were selected as the control group. The 2350 patients were assigned into group A and B based on the administration with narrow-spectrum antibiotic or broad-spectrum antibiotic. The feces specimens of patients at the 1st, 5th, 9th and 14th day after antibiotic treatment were collected to analyze the bacteriological data and the cases of intestinal flora imbalance after applying the narrow-spectrum and broad-spectrum antibiotic were compared and the differences in the bacterial colony compositions of intestinal floras from those of the healthy hypertension patients at the same period were analyzed.

**Results:**

The ratio of intestinal flora imbalance was 50.4% after applying antibiotic in patients from group A and 78.3% in group B. grade I and II imbalance were predominant in group A and grade III imbalance was the most severe one in group B (*P*<0.05). Compared with the intestinal flora in healthy elderly hypertension patients, the ratio of the primary composition flora of patients with imbalanced intestinal flora was changed obviously.

**Conclusion:**

The application of narrow-spectrum antibiotic and shortening the application time of antibiotic can more effectively protect the normal intestinal flora of elderly hypertension patients.

## Introduction

With the rapid development of modern medical technology, antibiotic has been extensively applied in various medical treatments. The dysfunction of intestinal flora and damage of the bacterial species and the number of the flora induced by the application of antibiotic are more obvious in elderly people (1, 2). The damage of intestinal flora may lead to a series of diseases of digestive tract like constipation and diarrhea and worse, directly cause cardiovascular diseases, threatening the life and health (3, 4).

The intestinal flora in normal people is mainly distributed in the colon, mainly consisting of the probiotics like lactobacillus and bifidobacterium (5, 6). Currently, studies on the intestinal flora imbalance are exploratory and hypothetical.

The present study aimed at investigating whether there are differences in the effects of antibiotic on the intestinal flora of elderly hypertension patients with infectious diseases and whether the differences are significant, with the expectation of providing references and suggestions for future treatment of elderly hypertension patients with infectious diseases.

## Materials and Methods

### Test objects

A total of 2350 hypertension patients with infectious diseases treated by antibiotic in Ordos Central Hospital (Inner Mongolia, China) from January 2010 to August 2016 were selected. Of them, there were 1376 male and 974 female cases and the average age was 64±0.4 yr old. A total of 1340 patients treated by narrow-spectrum antibiotic (penicillin, cephazolin, vancomycin, etc.) were assigned into group A with 768 male and 572 female cases; 1010 patients treated by broad-spectrum antibiotic (chloramphenicol, aureomycin, oxytetracycline, tetracycline, thiamphenicol, erythromycin, etc.) were assigned into group B with 60 male and 402 female cases. A total of 750 healthy hypertension patients who received physical examinations were selected as the control group with 430 male and 320 female cases and the average age was 62±0.5 yr old.

### Inclusion and exclusion criteria

Inclusion criteria: All the patients were the elderly patients with infectious diseases who were definitely diagnosed by the emergency department of our hospital; all the patients had hypertension concurrently; antibiotics were not applied in treating the infectious diseases prior to admission. Exclusion criteria: Disabled patients; patients with several complications; patients with intestinal flora imbalance prior to antibiotic treatment in Ordos Central Hospital; bedridden patients for a long term. All the test patients signed the informed consent.

The study was approved by the Ethics Committee of Ordos Central Hospital.

## Methods

The bacteriological analysis results of the feces specimens collected at the 1st, 5th, 9th and 14th day after antibiotic application were interpreted. The cases of patients with imbalanced intestinal flora were counted and the differences in the intestinal flora composition were analyzed by comparison with that of healthy control group. Referring to the 2016 International Grading Standard of Examining Intestinal Flora Imbalance by Feces Smear (7), the patients with imbalanced intestinal flora from both groups were graded and the differences caused by the application of narrow-spectrum and broad-spectrum antibiotic were compared. The effects of antibiotic on the intestinal flora in elderly hypertension patients with infectious diseases were analyzed and investigated.

The test reports of feces smears on the 1st, 5th, 9th and 14th day of patients from both groups were inspected and the standard of flora imbalance by feces smear (8) was used to evaluate the intestinal flora imbalance.

### Evaluation criteria

According to 2016 International Grading Standard of Examining Intestinal Flora Imbalance by Feces Smear (7, 8), the standard for each grade of the intestinal flora imbalance was as follows: grade I intestinal flora imbalance: The total bacterial number was lower than the normal value; no significant difference was seen by smear test; G+ bacilli number was normal; G− bacilli number was slightly increased and G+ cocci number showed no abnormal changes. Grade II intestinal flora imbalance: The total bacterial number was decreased obviously; a small number of white cells were seen by the smear test; G− bacilli number was increased significantly and G+ bacilli number was decreased and G+ cocci number showed no abnormal changes or normal increase. Grade III intestinal flora imbalance: The total bacterial number was decreased severely; a large number of white cells were seen by the smear test; one kind of bacterium or fungus was increased significantly and other original bacteria were decreased.

### Statistical methods

SPSS 23.0 software (Chicago, IL, USA) was used for the data analysis. The enumeration data were represented by rate and χ^2^ test was used for the comparison between groups. *P*<0.05 was considered as statistically significant difference.

## Results

### Results of clinical data comparison

In the clinical data comparison, gender, diarrhea, sleeping habits, activity habits, drinking and smoking, diet habits and clinical indicators showed no significant effects on the intestinal flora imbalance with no statistical differences (*P*>0.05) (Table 1 and 2).

**Table 1:** Comparisons of the clinical data of the patients from both groups

***Clinical Data***	***A***	***B***	***P***
Gender			0.516
Male	768 (57.3%)	599 (59.3%)	
Female	572 (42.7%)	411 (40.7%)	
Activity habit			0.329
Yes	637 (47.5%)	431 (42.7%)	
No	703 (52.5%)	579 (57.3%)	
Bad habits like drinking and smoking			0.207
Yes	899 (67.1%)	684 (67.7%)	
No	411 (32.9%)	326 (32.3%)	
Diet habit			0.285
Light	762 (56.9%)	467 (46.2%)	
Spicy	578 (45.1%)	543 (53.8%)	
Diarrhea within 1 month			0.364
Yes	5991 (44.7%)	534 (52.9%)	
No	741 (55.3%)	476 (47.1%)	
Sleeping habit			0.326
Sleep early	823 (61.4%)	655 (64.9%)	
Sleep late	517 (38.6%)	355 (35.1%)	
Clinical indicator			
Body temperature	38.82±0.57 °C	38.56±0.63 °C	0.293
White cell counts	(12.68±1.36) ×109/L	(11.75±1.71) ×109/L	0.326
Blood platelet counts	(121.0±2.0) ×109/L	(103.0±2.0) ×109/L	0.299
Hemoglobin counts	103.0±2.0 g/L	111.0±1.0 g/L	0.346
Procalcitonin	0.71±0.13 ng/L	0.86±0.08 ng/L	0.276

**Table 2:** The clinical data of the healthy control group

***Gender***	***Male***	***Female***
Activity habit	430 (57.3%)	320 (46.7%)
	Yes	No
Bad habits like drinking and smoking	472 (62.9%)	278 (38.1%)
	Yes	No
Diet habit	389 (51.9%)	361 (48.1%)
	Light	Spicy
Diarrhea within 1 month	398 (53.1%)	352 (46.9%)
	Yes	No
Sleeping habit	269 (35.9%)	481 (64.1%)
	Sleep early	Sleep late
	427 (57.0%)	323 (43.0%)
Clinical indicator		
Body temperature	36.72±0.88 °C	
White cell counts	(7.13±1.66) ×109/L	
Blood platelet counts	(196.0±2.0) ×109/L	
Hemoglobin counts	135±1.0g/L	
Procalcitonin	0.34±0.17ng/L	

### Cases of flora imbalance at different time

Patients with intestinal flora imbalance in group B using broad-spectrum antibiotic were much more than those from group A using narrow-spectrum antibiotic; in group A, on the 5th day, there was the largest number of patients with the intestinal flora imbalance, while in group B, there was the largest number of patients with the intestinal flora imbalance on the 5th day and the 14th day (*P*<0.05) (Table 3).

**Table 3:** Cases of flora imbalance on 1st, 5th, 9th and 14th day of both groups

***Time***	***A***	***B***	***P***
1d	62 (4.6%)	86 (8.5%)	0.052
5d	236 (17.6%)	245 (24.3%)	0.046
9d	189 (14.1%)	192 (19.0%)	0.057
14d	189 (14.1%)	268 (26.5%)	0.031
Total	676 (50.4%)	791 (78.3%)	0.038

### Flora composition ratio of patients with intestinal flora imbalance and healthy patients

The feces smears of intestinal flora of the healthy elderly hypertension patients and imbalanced patients were compared and the ratios of the primary composition flora in two groups were changed obviously. The differences presented statistical significance (*P*<0.01). The Gram positive bacilli in patients with antibiotic application was obviously lower than that of the healthy control group; the difference of the Gram negative bacilli between two groups was the largest and the intestinal flora imbalance induced the production of a large number of pathogenic Gram negative bacilli (like *Escherichia coli, Salmonella, Shigella*, etc.); Gram positive cocci was slightly increased; Gram negative cocci was slightly decreased (Table 4).

**Table 4:** Flora composition ratio of the patients with intestinal flora imbalance and healthy patients

	***Probiotics (%)***	***G+ bacilli (%)***	***G− bacilli (%)***	***G+ cocci (%)***	***G−cocci (%)***
Patients with intestinal flora imbalance (n=1467)	11.77	62.18	75.45	16.77	1.12
Healthy control group (n=720)	23.68	6.59	22.46	8.25	7.20
P	0.008	0.004	0.005	0.009	0.009

### Intestinal flora imbalance ratio of elderly hypertension patients after applying antibiotic

In all the patients, intestinal flora imbalance occurred in 1467 cases, accounting for 62.4%. Among them, the flora imbalance ratio of patients from group A using narrow-spectrum antibiotic was 50.4% (676 cases). Overall, 236 cases were in grade I, accounting for 34.9% of the total patients with intestinal flora imbalance; there were 328 grade II cases, accounting for 46.2% of the total patients with intestinal flora imbalance; there were 128 grade III cases, accounting for 18.9%. The flora imbalance ratio of patients from group B using broad-spectrum antibiotic was 78.3% (791 cases), which was the highest. Among them, there were 28.4% (225) grade I cases; there were 33.9% (268) grade II cases; there were 49.2% (389) grade III cases. All the differences presented statistical significance (*P*<0.05) (Table 5).

**Table 5:** Intestinal flora imbalance ratio of elderly hypertension patients after applying antibiotic

***Group***	***Grade I intestinal flora imbalance***	***Grade II intestinal flora imbalance***	***Grade III Intestinal flora imbalance***	***Flora imbalance case/total case***
Group A	236 (34.9%)	328 (46.2%)	128 (18.9%)	50.4%
Group B	225 (28.4%)	268 (33.9%)	389 (49.2%)	78.3%
*P*	0.034	0.041	0.027	0.030

## Discussion

Tens of thousands of microorganisms inhabit the human bodies and among them, the intestinal flora plays a vital role in the regulation of the physical functions and maintenance of the physiological balance (9, 10). The intestinal flora, consisting of 99.9% anaerobic bacteria, includes multiple normal floras and sensitized floras (11, 12). For elderly people, especially those with hepatic and renal hypofunctions, antibiotic metabolism weakening and long-term medications, the intestinal flora is dramatically decreased due to the inhibition of the normal number, leading to the intestinal flora imbalance (13, 14); even a series of complications such as diabetes (15) and toxemia (16), which directly endangers the life.

At present, it is a very important topic that whether the application of a large number of antibiotics could have a certain or a relatively severe effect on intestinal floras of the human, especially the elderly people. According to the clinical practice reports, there are no related studies on the effects of the antibiotic on elderly hypertension patients with infectious diseases. In the present study, the retrospective analysis was used and a total of 2350 representative elderly hypertension cases with infectious diseases were selected to make comparisons. The experimental results have a certain reference and guiding significance for the clinical studies and application of the antibiotic.

In comparisons of clinical data, the gender, diarrhea, sleeping habits, activity habits, drinking and smoking, diet habits and clinical indicators had no effects on the intestinal flora imbalance. This is similar to the study by Tang T et al. (17). Among patients using the narrow-spectrum antibiotic from group A, there were 676 cases with the intestinal flora imbalance in total in 14 days and the number of patients who had the intestinal flora imbalance on the 5th day was the largest, accounting for 17.6% of the total cases. Among patients using the broad-spectrum antibiotic from group B, there were 791 cases with the intestinal flora imbalance in total and compared with other time periods, on the 5th day and 14th day, there was the largest number of patients who had the intestinal flora imbalance. Through the comparison of the experimental subjects from two groups, the intestinal flora imbalance in group A using the broad-spectrum antibiotic were severer and the grade III flora imbalance accounted for 78.3%.

According to the experimental results, elderly hypertension patients with infectious diseases treated by the broad-spectrum antibiotic are more vulnerable to the intestinal flora imbalance than those treated by the narrow-spectrum antibiotic. The reason is the long-term application of the broad-spectrum antibiotic disturbs the normal intestinal flora and destroys the biological screen; the majority of pathological bacteria overgrow and the normal floras are mutated quantitatively or qualitatively as a result, namely, the intestinal flora imbalance (18). The longer the application is, the severer the destruction caused to the intestinal flora of patients will be. The intestinal probiotics are the anaerobic bacteria, mainly consisting of *Bifidobacterium* and can be bound with the specific receptors on the surface of the intestinal mucosa to generate a layer of velum structure inhibiting the intestinal pathogenic bacteria (19).

For elderly patients, the physical hypofunction and the weakening of the autoimmune system capacity significantly increase the survival rate of the pathological bacteria in the intestinal flora. What is more, because the hypertension patients need to take antihypertensive medicines and occasionally are treated by combining with other medicines based on the disease conditions, it is inevitable that the adverse reactions of medicines occur and the flora resistance is generated. The ratios of bacteroides and firmicutes are greatly increased, but the number of the *Bifidobacterium* and *Lactobacillus* is decreased. As a result, the velum structure which can inhibit the pathologic bacteria in human bodies is severely damaged and a large number of harmful bacteria in intestinal tract are reproduced. Therefore, it is easier to suffer from the intestinal flora imbalance. Furthermore, there are significant differences in the intestinal flora composition between the elderly hypertension patents with infectious diseases and suffering from intestinal flora imbalance and healthy hypertension patients, and for the former, the main change is the decrease in the number of probiotics and the increase in that of pathologic bacteria. Because the killing of intestinal bacteria by antibiotic and the change of intestinal environment produce a combined effect and the probiotics, belonging to the sensitive flora are weakly resistant to the change of the environment, the number of the probiotics is decreased to a large extent (20).

The bacteriological analysis is the most typical testing method of the intestinal flora in the experiment. This can help to visually find the flora species and number in patients’ specimens (21, 22). However, using this method is hard to separate the unculturable bacteria in the specimens and only the culturable bacteria can be detected quantitatively. Moreover, the storage of specimens and misoperation in the experiment may lead to a certain error (23).

## Conclusion

For elderly hypertension patients with infectious diseases, the intestinal flora disturbance is severer in those treated by the broad-spectrum antibiotic. Elderly hypertension patients with infectious diseases should use the narrow-spectrum antibiotics as much as possible and shorten the application time of the antibiotic to protect the normal intestinal flora.

## Ethical considerations

Ethical issues (Including plagiarism, informed consent, misconduct, data fabrication and/or falsification, double publication and/or submission, redundancy, etc.) have been completely observed by the authors.
